# When patient advocacy organizations meet industry: a novel approach to dealing with financial conflicts of interest

**DOI:** 10.1186/s12910-019-0435-1

**Published:** 2019-12-17

**Authors:** Orna Ehrlich, Laura Wingate, Caren Heller, Inmaculada de Melo-Martin

**Affiliations:** 1 Crohn’s & Colitis Foundation, New York, NY USA; 2 Division of Medical Ethics, Weill Cornell Medicine, 407 E.61st St., RR-212, New York, NY 10065 USA

**Keywords:** Ethics, Ethics committee, Financial conflicts of interest, FCOI, Patient advocacy organization, PAO

## Abstract

**Background:**

Much like academic-industry partnerships, industry financial support of patient advocacy organizations (PAOs) has become very common in recent years. While financial conflicts of interest (FCOI) between PAOs and industry have received more attention in recent years, robust efforts to mitigate these conflicts are still limited.

**Main body:**

The authors outline the possible benefits and ethical concerns that can result from financial interactions between biomedical companies and PAOs. They argue that the use of novel strategies, such as the creation of a standing ethics committee, could be helpful in managing FCOIs and ensuring the warranted trust of PAO’s constituents. Although ethics committees to address FCOIs are common in the academic context, its use by PAOs is still limited. The authors conclude by describing the process of development and implementation of such an ethics committee at the Crohn’s & Colitis Foundation.

**Conclusions:**

While collaborations with industry can result in conflicts of interest, PAOs can develop strategies to address those conflicts. One such strategy is the creation of a standing independent ethics committee to guide PAOs on new and/or existing programs and protocols as they pertain to their industry relationships.

## Background

Academic-industry partnerships have become central to producing advances in biomedical and clinical research and to bringing such advances to clinical practice [1–3]. Partnerships among pharmaceutical, device, and biotechnology industries, and physicians and scientists can encourage innovative approaches necessary to address complex health problems. They can also provide financial resources in the face of limited governmental and nonprofit funding. Nonetheless, such collaborations come with some costs. Among a variety of the concerns that these relationships create, the presence of financial conflicts of interest (FCOI) not just for individual researchers and clinicians but also for institutions has received a significant amount of attention [4–7]. An institutional conflict of interest occurs when an institution’s secondary interests or those of its senior officials create risks of undue influence on decisions involving the institution’s primary interests [5]. Various studies have found that FCOI can adversely affect the objectivity and integrity of research and can compromise the clinical care of patients [8–11]. Concerns about these negative effects has led academic institutions, federal agencies, and publishers to develop policies to attempt to mitigate such effects. Conflicts of interest policies usually include three distinct, but related, elements: disclosure of conflicts; management of the conflicts that are thought to be significant; and prohibition of activities when such is thought to be necessary to protect the public interest [5–7].

Academic institutions are not the only ones where collaborations with industry are raising ethical concerns. Patient advocacy organizations (PAOs), whose primary goal is generally to address some particular disease or disorder through advancing research and/or improving the quality of care for patients and families affected by those medical conditions, are increasingly entering into financial arrangements with biomedical industries [12–16]. According to a recent study, industry financial support of PAOs is very common, with at least 83% of 104 of the largest patient organizations in the U.S. receiving financial support from drug, device, and biotechnology companies [13]. Although evidence suggests that industry support of PAOs is quite variable, it is often significant. Some studies have shown that at least 39% of the organizations assessed received at least $1 million annually from industry [13]. Another study evaluating 289 U.S. PAOs found that they received an average of 45% of their income from biomedical companies [14]. The situation seems to be similar internationally. For instance, evidence shows that interactions between PAOs and pharmaceutical companies are common in the United Kingdom, Germany, Italy, and Australia [17–19].

Collaborations between PAOs and industry are certainly of benefit to biomedical companies [20]. PAOs have a credibility that pharmaceutical companies not uncommonly lack. They are trusted not just by the patients and healthcare providers they serve but by government agencies, healthcare institutions, and members of the public at large. As it is the case with industry collaboration with academia or medical professionals, funding from industry can provide PAOs with needed resources to promote their primary mission. However, similar to industry relationships with physicians or academic institutions, interactions between industry and PAOs can be ethically problematic and can create FCOIs. Here we discuss some of the possible benefits and ethical concerns that can result from financial interactions between biomedical companies and PAOs. We also propose a novel strategy that PAOs could use to help manage some of the risks that these relationships involve.

### PAOs relationship with industry: the benefits

Collaborations with industry can result in a variety of opportunities for PAOs. Medical research and new drug discovery are expensive and time consuming. Evidence shows that getting a new candidate molecule from discovery to market requires $2.5 billion and over 10 years of investment [21]. This demonstrates not only the tremendous financial investment to develop new treatments but also the time and labor needed. Despite the rise in chronic diseases in the U.S. [22], the National Institutes of Health, one of the nation’s primary funders of medical research, has been relatively flat in its grant funding for the last 10 years [23]. Furthermore, federal research funding is limited and subject to dramatic shifts, depending on congressional agendas and presidential administration interests. Given the volatility of public funding, PAOs can function as efficient catalysts of research and successful collaborations [24].

Increasingly, organizations, such as the Crohn’s & Colitis Foundation, are investing more in research to meet their mission. They are providing funding for basic, translational, and clinical research, and are engaging with patients in the research process and the drug development process. While PAOs have focused historically on funding investigator-initiated grants at academic centers [24], in recent years, many of these organizations have begun to fund research on new drug targets and drug candidate molecules, with an emphasis on targets that would have struggled to make the leap from bench to bedside given the cost of new drug discovery. Additionally, PAOs are engaging with patients to determine which outcome measures are important to them to include in clinical research and to understand which attributes of clinical trials would influence their decision to participate. Some of them are also setting up patient registries for longitudinal follow-up through patient-reported outcomes, clinical reports, and biosamples.

This investment in research requires financial resources. Partnering with industry on drug discovery and development allows PAOs to fund research projects that can be of benefit to patients and caregivers. Moreover, such partnering permits PAOs to further investigations, while also engaging patients in various aspects of the research process, from bringing their voices to prioritization of disease outcomes and meaningful clinical trial characteristics, to promoting enrollment in specific clinical trials.

PAOs are also well positioned to educate stakeholders and advocate on their behalf to improve quality of life and access to medical and other types of interventions. However, programs have shifted from live to web-based education and support. For instance, according to some studies, 72% of consumers claim the use of technology is important when it comes to managing their health [25]. This is even true in underserved areas where traditional care delivery models struggle and where internet health education programs are helping to bridge communication gaps and activate patients. Researchers and experts expect increased use of the internet in disease education [26]. As with PAOs involvement in the research process, all these changes have increased PAOs’ need for financial resources so that they can fulfill their goals of education and support for patients and families. Industry funding, together with grants and sponsorship from foundations, and individual donors can help PAOs achieve this goal.

Another way in which PAOs benefit from collaborating with industry relates to their power in convening stakeholders. Many PAOs have missions that call for driving innovation to tackle the lack of effective treatments or diagnostics, addressing the high-cost of drugs and access, and educating and supporting the patient and provider communities. For them to be successful, all of these activities require the involvement of various parties, from academia, industry, and regulatory agencies, to insurers, health systems, and patients. PAOs are well suited to bring these groups, with differing and sometimes conflicted points of view, together for discussion. But the ability to convene all relevant stakeholders at the table also requires financial resources. Collaborations with industry can contribute to PAOs ability to meet these aspects of their missions.

### PAOs relationship with industry: the concerns

Although partnerships between industry and PAOs can help PAOs advance their missions, these collaborations can also create institutional FCOI and thus raise ethical concerns. A PAO’s primary mission is to serve the interests of its stakeholders, primarily people with the disease or condition on which the PAO focuses and those of its families and caregivers. As mentioned, to achieve this mission, PAOs need funds. Collaborations with industry pose risks that these financial needs will unduly influence PAOs’ decisions in ways that conflict with their primary interest of serving the needs of their patient- and caregiver-constituencies. These conflicts are made more salient when a significant number of PAOs have a current or former industry executive on the governing board and/or are in a position to influence decisions affecting public policy on healthcare [13].

Such undue influence can affect various aspects of the organization’s mission [13, 14]. For instance, as we indicated above, PAOs offer direct counseling and education to their patients by providing them with help centers, brochures, web pages, and live and online programming. Drug and device companies have an interest in selling their products. Thus, when they fund PAOs educational activities, such funding can potentially compromise the neutrality of the information patients receive. Many PAOs fund or conduct medical and health services research. Funding by biomedical companies may influence these activities in ways that serve the companies’ interests more strongly than those of the patients and caregivers the PAO serves. Likewise, PAOs are sometimes involved in clinical trial efforts, including recruitment, which can pose an FCOI if they interact with industry on these efforts. Financial dependence on pharmaceutical or device industries can also lead PAOs to promote the use of costlier or less effective drugs. When competing medical products are available, funding from some companies might lead PAOs to favor the interventions of one company over others regardless of their benefits and risks [27]. Similarly, PAOs often engage in policy advocacy and can exert significant influence in shaping legislative and research agendas [17]. Collaborations with industry can negatively affect these efforts and may skew a PAO’s actions. For instance, it can lead them to seek quicker approval of medical interventions and thus put patients’ health at risk from inadequately tested drugs or devices [28]. Likewise, FCOIs can direct PAOs to advocate for insurance coverage of interventions that might offer little help to patients and families. These concerns are made more significant when considering inequalities in the funding of PAOs. Industry might have more interest in collaborations with some PAOs rather than others, which might lead more heavily founded PAOs to exert a greater influence on research, policy, and/or clinical practice.

Even if FCOIs do not have a negative influence on the PAO’s mission, the existence of such conflicts can create the appearance of bias and erode the trust that patients and families, public agencies, healthcare institutions, and the public at large place in PAOs. If trust is damaged, patients and families can doubt the reliability of education and information provided by the PAO. Public agencies and healthcare institutions could question the credibility of PAOs and ignore their advice. And the public might be less supportive of PAO activities if they believe that industry funding is biasing their advocacy or capturing their agenda. Some evidence suggests that members of the public are more suspicious of the influence of vested interest when research is funded by industry [29]. Some data about people’s attitudes toward conflicts of interest in medicine and biomedical research also shows that people believe that the existence of conflicts of interest decreases the quality of the research evidence [30, 31]. Hence, when assessing the risks and benefits of PAO involvement with industry, it is important to take into account not only the risk that conflicts might bias PAO’s various advocacy activities, but also the risks that perceptions of bias, whether present or not, can lead to a loss of trust in the PAO.

In spite of these concerns, some evidence suggests that PAOs might not be attending sufficiently to the problems that arise when they enter into financial relationships with biomedical companies [13, 15, 16, 19, 20, 32, 33]. Few of these organizations have published FCOI policies, which does little to assuage the possible worries that patients, families, healthcare institutions, public agencies, and the public might have regarding the ability of PAOs to promote their primary missions. Indeed, according to some evidence, PAOs’ disclosures of their relationships with industry, one of the basic aspects of conflicts of interest policies, are far from ideal [13, 15, 17, 33]. For instance, some studies have shown that the disclosure practices of many of these organizations are limited, with many of them publishing the names of the industry funders but with few disclosing the amounts provided or their use [13, 14]. This can lead to further distrust of PAOs as stakeholders might come to believe that these organizations are attempting to hide their relationships with industry.

Of course, disclosure of conflicts is only a first step in the direction of addressing them, as disclosure of commercial relationships does nothing to address the conflicts in question and might actually contribute to making the effects of those conflicts worse [34, 35]. Hence, in addition to promoting transparent industry-PAO collaborations, other strategies are needed to ethically manage the conflicts that result from such partnerships.

### A novel strategy to address FCOIs: creating an ethics committee

Some best practice recommendations aim at helping PAOs to deal with financial conflicts of interest [36]. For instance, publicly reporting the sources of revenue received and encouraging a diversity of revenue sources, including individual donations, bequests, corporate sponsorships, foundation funding, and government grants, sponsorships, and contracts. In addition, PAOs have investment income and many have a membership or dues program that generate additional revenue. A diverse revenue model ensures sustainability for PAOs and provides protections from market variations [37]. It can thus limit the possible negative effects of financial collaborations with industry, as PAOs need not rely solely on this support. Furthermore, the existence of PAO volunteers, stakeholder, and various watchdog groups also contribute to PAOs’ accountability.

Nevertheless, as discussed above, increasing collaborations between PAOs and industry raise concerns about how an organization funds research, provides education, support, and advocacy while ensuring that its work attends to the needs of its patients and caregivers. With limited successful approaches described in the literature, our goal in this paper is to provide an overview of a novel strategy for PAOs to consider to address this need, specifically a standing ethics committee. Although ethics committees to address FCOIs are common in the academic context, its use has not been sufficiently explored by PAOs.

Like other PAOs, the Crohn’s & Colitis Foundation has developed a deep relationship of trust with patient and provider communities involved in inflammatory bowel diseases (IBD) over the past 50 years. As the Foundation strives to be a convener and catalyst to achieve its mission to cure Crohn’s disease and ulcerative colitis and improve the lives of those affected by these diseases, new initiatives and partnership opportunities, some of them with industry, have emerged. Cognizant of the fact that these collaborations can create FCOIs, and wanting to ensure the reliability of its clinical, research, and educational programs and initiatives and safeguard and nurture warranted trust, the Foundation decided to bring independent expertise to guide it through these considerations in a systematic manner. Thus, the Foundation established a standing ethics committee in 2015.

An ethics committee charged with assessing collaborations with industry can provide input and guidance on PAOs’ programs and policies regarding interactions with patients, providers, and sponsors, so as to ensure that any conflicts of interest in the activities performed by the Foundation are appropriately managed. For the Crohn’s & Colitis Foundation, the committee advises on issues related to human subjects protections, such as the appropriate use of biosamples and patient data, confidentiality of participants, measures to ensure protection of patient privacy, and mechanisms for recruitment of research participants (e.g., honoraria, approaching participants, biosamples (if used), and any conflicting roles of those participating in these activities). The ethics committee also guides the Foundation on the level or type of involvement of a financial supporter that might be ethically sound. It thus considers and advises on whether such collaborations are ethically appropriate during the planning, implementation, delivery, and review stages of projects. It advises on the development of policies for collaborations with industry as well.

Two considerations are particularly important when contemplating the creation of an ethics committee charged with evaluating relationships with industry: diversity of expertise and independence of the committee. Given the various aspects of relevance when assessing FCOIs, PAOs’ committee membership should be comprised of various stakeholders who can bring pertinent expertise. For the Crohn’s & Colitis Foundation this includes medical leadership, Foundation board members, patients/caregivers representatives, and independent experts, including a bioethicist, a research methods expert, and an academic clinical investigator. An independent bioethicist (IdMM) chairs the Committee, with support by a Foundation staff member (OE) who has no relationship with the programs/initiatives the Committee evaluates, but who is knowledgeable of the innerworkings of the Foundation.

**Fig. 1:**
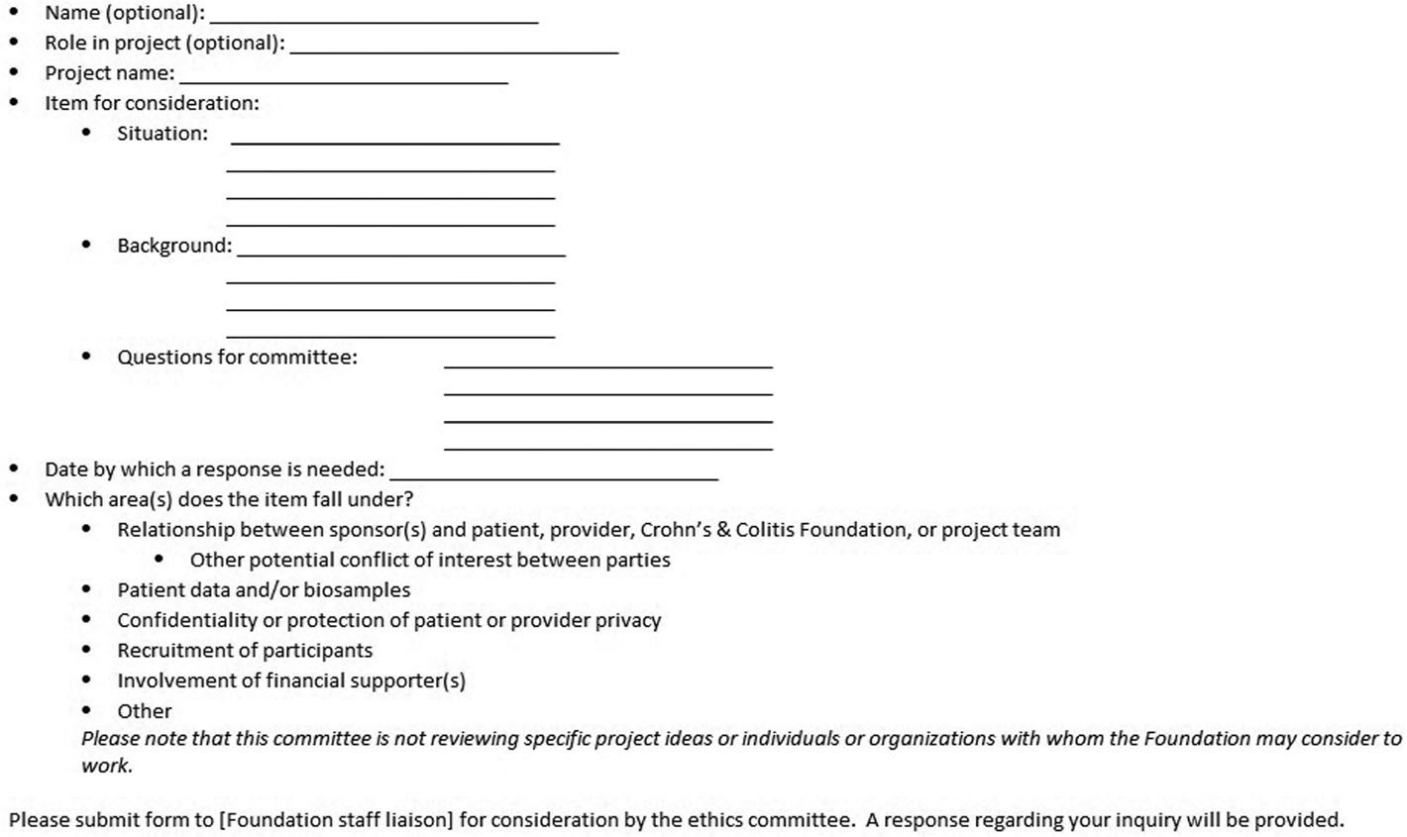
Submission form for Crohn’s & Colitis Foundation ethics committee

Procedurally, any staff or constituent can submit an ethics committee review form (see Fig. 1), which is evaluated by the committee chair to determine whether the request needs a full committee review or whether the chair can provide a recommendation on the inquiry. Although this might limit the effectiveness of the committee in managing conflicts, the fact that staff members are committed to the importance of the committee’s role and that they believe the committee’s work enhances their programs by contributing to nurturing the trust of patients and caregivers, makes this concern less pressing. When necessary, the Committee’s chair has conversations with program managers to obtain all relevant information. Since its formation in 2016, the Committee has convened several times by teleconference to evaluate review requests. After discussions, the chair writes a report providing a recommendation as well as the reasons for it. The Foundation staff liaison then disseminates the response to the appropriate project lead(s) and team(s) for their consideration. Should an ethics breach occur, the committee will be notified and an ad hoc meeting will be convened to discuss mitigation, resolution, and steps to prevent for future instances.

**Fig. 2:**
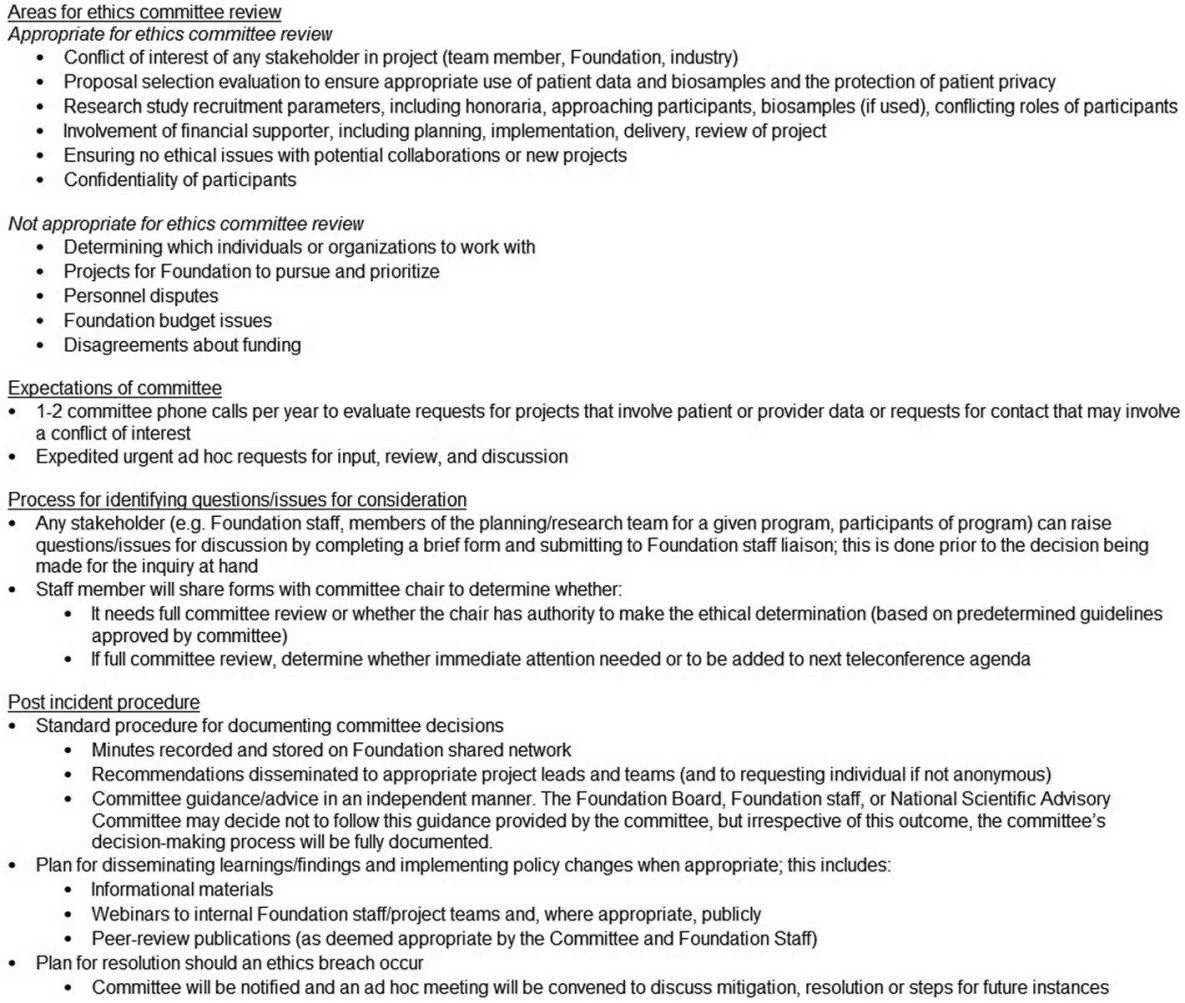
Overview of Crohn’s & Colitis Foundation ethics committee role process, and procedure

What the authority –binding or advisory-- of the ethics committee is will depend on a PAOs’ needs, organizational structure, and so on. In most cases, these committees are likely to have an advisory role. Such is the case for our Foundation’s ethics committee, which advises the Foundation but does not determine the types of projects the Foundation pursues or prioritizes or the individuals or organizations with whom the Foundation will work. Nonetheless, the Foundation’s leadership is deeply committed to the importance of the Committee and takes the Committee’s evaluations and recommendations seriously when making the final determination on how best to proceed with the inquiry at hand. Figure 2 includes a more detailed overview of the committee’s role, the process that is followed for review and post-decision steps, including steps for disseminating policies changes when appropriate.

To date the committee has reviewed 17 requests and advised the Foundation about important decisions related to various collaborations that raise FCOIs concerns. Examples of ethics committee reviews include guiding the Crohn’s & Colitis Foundation on royalties and intellectual property guidelines, advising on social media and parameters for brainstorming potential partnership ideas with a new company, providing parameters for how the Foundation should interact with companies with clinical trials wishing to engage with the Foundation’s Clinical Trials Finder, and providing ethical considerations for establishing a new program as well as ensuring the Foundation upholds our peer review integrity when new program opportunities arise. The Committee has also advised on concerns related to educational programs, such as reviewing existing policies and processes on transparency in listing funding sources in program materials as well as our annual report.

Of course, the creation of institutional ethics committees to address financial conflicts of interest in the context of PAOs is not without objection. Like ethics committees in other contexts, e.g., in hospitals or research institutions, they raise concerns about true independence. Even if the members of the Committee are independent of the particular PAO, Ethics Committees are ultimately institutional committees concerned also with protecting the interest of the organization. This might lead members to discount some problems or minimize them. Nonetheless, it is not clear that any other strategy would be free of this concern. Even if these Ethics Committees were to be freestanding commercial committees –as it is the case with some Institutional Review Boards, they would still be dependent on their clients. Similarly, if the PAOs’ boards developed their own policies and made decisions themselves these concerns would still be present.

A related worry about the creation of Ethics Committees to manage financial conflicts is that they can serve as “seals of approval” that contribute to the reputation of the PAO, without actually having any authority regarding the management of financial conflicts. Although the existence of Ethics Committees might lead to the incorrect believe that conflicts of interest are being managed in appropriate ways when they are not, the possibility of this misperception does not seem sufficient to outweigh the potential benefits of these committees. Safeguarding the Committees’ independence can help assuage this concern. Also important to minimize this problem will be to ensure that the PAO’s senior leadership is supportive of the Committees’ efforts and encourages their employees to utilize it and incorporate their guidance into decisions. Likewise, a transparent process of review can also mitigate this concern. Ethics committee’s recommendations could be available to staff, volunteers and relevant stakeholders as a way to promote warranted trust. Given these potential drawbacks, it is important for PAOs to consider an ethics committee in addition to other solutions to mitigate FCOIs, such as developing conflicts policies and ensuring transparency.

## Conclusions

Collaborations with industry can result in conflicts of interest, but PAOs can develop strategies to address those conflicts either by managing them when they exist or by eliminating the conflict. The Crohn’s & Colitis Foundation has addressed this need in a novel and proactive way: the creation of a standing ethics committee. Although ethics committees to deal with conflicts of interest concerns are common in other contexts, e.g., academic institutions, we are unaware of other PAOs with a similar committee.

There are several keys to the effectiveness of our ethics committee that can be considered for other PAOs interested in establishing their own committee. First, it is led by an independent bioethicist with expertise on normative ethics and the biomedical sciences, and who has done a significant amount of work on issues related to conflicts of interest. Thus, PAOs should take strides to find members with ethics expertise to serve as the lead of their committee. Second, the committee recommendations attend to the need to promote the welfare of patients and nurture the warranted trust of relevant stakeholders, while acknowledging the legitimate needs of the Foundation to collaborate with industry to advance its mission. This is important for PAOs to keep in mind as they establish the guiding principles of their ethics committee and the process by which they review submissions. Third, the Foundation staff who advance the research, education, support, and advocacy activities are strongly committed to promoting the interests of patients, caregivers, and relevant stakeholders and protecting the trust that they place in the organization. They thus have embraced the ethics committee and they welcome the opportunity to bring issues to it. Relatedly, but particularly important to point out, the Foundation’s leadership is committed to following the ethics committee’s advice to mitigate the negative effects of the conflicts under consideration, even if this action can result in loss of revenue. Thus, ensuring that both staff and leadership are on board with the creation and charge of an ethics committee is particularly important to optimize its effect and to assuage concerns that Ethics Committees might simply be a strategy to merely appease those worried about the negative effects of conflict of interest.

Of course, we recognize that the creation of an ethics committee might not be feasible for all PAOs and that it will not solve all of the concerns that financial conflicts of interest raise. Nonetheless, the expertise and diverse perspectives that an independent committee can provide as well as our experience with it suggests that it could be beneficial to other PAOs as well when confronting FCOIs in a proactive manner. The Foundation’s experience and processes described here can be used as a guide for other organizations interested in pursuing this approach.

## Data Availability

Not applicable.

## Abbreviations

FCOI: Financial conflict of interest
IBD: inflammatory bowel diseases
PAO: Patient Advocacy Organization
